# Lower leg symmetry: a Q3D-CT analysis

**DOI:** 10.1007/s00276-022-02940-9

**Published:** 2022-05-10

**Authors:** Gwendolyn Vuurberg, Jari Dahmen, Iwan G. G. Dobbe, Roeland P. Kleipool, Batur Hayat, Inger N. Sierevelt, Geert Streekstra, Gino M. M. J. Kerkhoffs, Sjoerd A. S. Stufkens

**Affiliations:** 1 Amsterdam UMC, Department of Orthopedic Surgery, Amsterdam Movement Sciences, University of Amsterdam, Meibergdreef 9, 1105 AZ Amsterdam, The Netherlands; 2 Academic Center for Evidence-Based Sports Medicine (ACES), Amsterdam, The Netherlands; 3 AMC/VUmc IOC Research Center, Amsterdam Collaboration on Health and Safety in Sports (ACHSS), Amsterdam, The Netherlands; 4 Amsterdam UMC, Department of Radiology and Nuclear Medicine, Amsterdam Movement Sciences, University of Amsterdam, Amsterdam, The Netherlands; 5 Department of Radiology and Nuclear Medicine, Rijnstate Hospital, Arnhem, The Netherlands; 6 Amsterdam UMC, Department of Medical Biology, Amsterdam Movement Sciences, University of Amsterdam, Amsterdam, The Netherlands; 7 Amsterdam UMC, Department of Biomedical Engineering and Physics, Amsterdam Movement Sciences, University of Amsterdam, Amsterdam, The Netherlands; 8 Specialized Centre for Orthopedic Research and Education (SCORE), Xpert Orthopedics, Amsterdam, The Netherlands

**Keywords:** Symmetry quantification, Deformity correction, Templating, CT, Tibiae, Lower leg, Osteotomy

## Abstract

**Purpose:**

In fracture and realignment surgery, the contralateral unaffected side is often used as a model or template for the injured bone even though clinically valuable quantitative data of bilateral symmetry are often unavailable. Therefore, the objective of the present study was to quantify and present the bilateral symmetry of the tibia and fibula.

**Methods:**

Twenty bilateral lower-leg CT scans were acquired in healthy volunteers. The left and right tibia and fibula were segmented resulting in three-dimensional polygons for geometrical analyses (volume, surface and length). The distal and proximal segment of the right tibia of each individual was subsequently matched to the left tibia to quantify alignment differences (translation and rotation). Bone symmetry on group level was assessed using the Student’s *t* test and intra-individual differences were assessed using mixed-models analyses.

**Results:**

Intra-individuals differences were found for tibia volume (5.2 ± 3.3 cm^3^), tibia surface (5.2 ± 3.3 cm^2^), translations in the lateral (*X*-axis; 9.3 ± 8.9 mm) and anterior direction (*Y*-axis; 7.1 ± 7.0 mm), for tibia length (translation along *Z*-axis: 3.1 ± 2.4 mm), varus/valgus (φ_z_: 1.7^o^ ± 1.4°), and endotorsion/exotorsion (φ_z_: 4.0^o^ ± 2.7°).

**Conclusion:**

This study shows intra-individual tibia asymmetry in both geometric and alignment parameters of which the surgeon needs to be aware in pre-operative planning. The high correlation between tibia and fibula length allows the ipsilateral fibula to aid in estimating the original tibia length post-injury. Future studies need to establish whether the found asymmetry is clinically relevant when the contralateral side is used as reference in corrective surgery.

**Level of evidence:**

III cohort study.

## Introduction

In fracture treatment and the correction of symptomatic malunions or other acquired deformities (for example as late complications of trauma), an anatomical reconstruction is considered of paramount importance for good clinical outcome [1, 2]. Additionally in knee arthroplasty, ankle arthrodesis and arthroplasty adequate alignment may be essential to restore function. Physicians often use the unaffected contralateral side of the patient as a rough reference standard in pre-operative planning although precise knowledge may further improve functional outcome [3–11]. From an anatomical and embryological perspective, assuming symmetry in development of the upper and lower extremities, using the contralateral unaffected side as a template may be an accurate option, though its validity is an assumption rather than a given fact [12–14]. A hypothesis explaining observed bilateral asymmetry originates from asymmetrical development that finds its genesis from the prenatal stage up to maturity [12, 15–17]. This asymmetry may also be explained by differences in mechanical stress during development, which in turn may cause kinematic asymmetry [18].

Detailed knowledge on anatomy and thus bone geometry is essential to restore function. For the ankle joint, not only the tibia is of paramount importance for optimal function, but also the fibula is essential to ensure joint congruency and prevent (sub) luxation of the talus [19]. In addition to providing stability to the ankle joint during a great range of mobility tasks, the fibula may help in correction surgery when it concerns correction of tibia bone length (comparable to the radius and ulna) [20].

Bilateral bone asymmetry is a current topic of interest for those who perform surgical corrections. Studies on bone asymmetry of the lower extremity have predominantly focused on the femur and tibia [12, 13, 15, 21–25]. Previously conducted studies have some limitations that make the results less generally applicable, such as comparison of injured with uninjured individuals, usage of cadaveric specimens with an unknown medical (lower-extremity) history, and a low number of subjects being studied (i.e., < 10). Moreover, none of the previous studies included a full three-dimensional in vivo analysis of geometrical and alignment differences of the tibia within healthy individuals (right–left differences) nor translated significant findings to implications for surgical practice.

The objective of the present study is therefore to quantify the bilateral asymmetry of the tibia in healthy volunteers using a computed tomography (CT-) scan and advanced 3D image analysis techniques.

## Materials and methods

High-resolution lower-leg CT scans were acquired in twenty healthy volunteers. Subsequently, 3D polygons were created and used to quantify intra-individual tibia differences in terms of geometry (volume, surface area and length) and anatomical alignment (translation and rotation) of the distal tibial segment with respect to the proximal segment of the tibia. Fibula polygons were also created to investigate its length in relation to tibia length.

### Study population

To ensure a balanced sample from the general population and to investigate the effect of gender specific characteristics, 10 males and 10 females were included [26, 27].

Prior to inclusion, volunteers were screened for health (i.e., no systemic diseases and no previous trauma to the lower leg requiring medical attention). The volunteers had to be at least 18 years old to provide consent for participation. To minimize the radiation load, volunteers were excluded if they had undergone a CT scan in the previous year, or were planned to undergo a CT scan in the upcoming year. Volunteers with musculoskeletal complaints of the lower extremity at the time of scanning were excluded. Additionally, females were excluded if they were pregnant or wanted to become pregnant.

Informed consent was obtained from all individual volunteers before inclusion in the study. Of each included volunteer sex, age, weight, body height and leg dominance were recorded.

### Ethical approval

All the procedures performed in this study involving human participants were in accordance with the ethical standards of the institutional research committee and with the 1964 Helsinki declaration and its later amendments (reference: 2017_039#B2017175a, approval date 24-03-2017).

### Image acquisition

Of each volunteer, a bilateral CT scan was carried out of the knees, lower leg and feet using a Brilliance 64 CT scanner (Philips, Amsterdam, The Netherlands) (voxel size 0.46 × 0.46 × 0.45 mm, 120 kV, and 160 mAs) [28].

### Creating virtual bone models

All CT scans were analyzed for symmetry in the same way as described by Vroemen et al. with proven excellent reliability (ICC 0.81–0.94) [20, 29]. In short, both tibiae and fibulae were segmented by threshold-connected region growing followed by a binary closing algorithm for filling of residual holes and closing of the outline [4]. A Laplacian level-set segmentation growth algorithm was subsequently used to grow toward the boundary of the bone. The Marching cubes algorithm was then used to extract a polygon mesh at the zero level of the level-set image. The polygon meshes represented the virtual 3D surface models of the bones.

### Bone symmetry parameters

Primary outcome measures included geometric and alignment parameters for defining bone symmetry expressed by parameters representing (absolute) intra-individual differences. To evaluate if tibiae were symmetrical, geometric parameters of left and right tibiae were statistically compared on both group level and within individuals. The relative difference in geometric parameters was calculated based on the percentage difference between the mean intra-individual difference and mean geometric parameter. The relative difference in translation parameters was calculated based on the mean intra-individual difference in relation to the axis length (in percentages). As secondary outcome measures, we evaluated if symmetry parameters were related to gender or leg dominance.

#### Geometric parameters

Based on the polygon meshes of the tibiae, the following geometric parameters were automatically calculated: volume (cm^3^), surface area (cm^2^) and length (cm). The bone surface represented the cortical bone surface. A difference in bone length was represented by translation along the Z-axis of the distal bone segment with respect to the proximal bone segment.

#### Alignment parameters

**Fig. 1:**
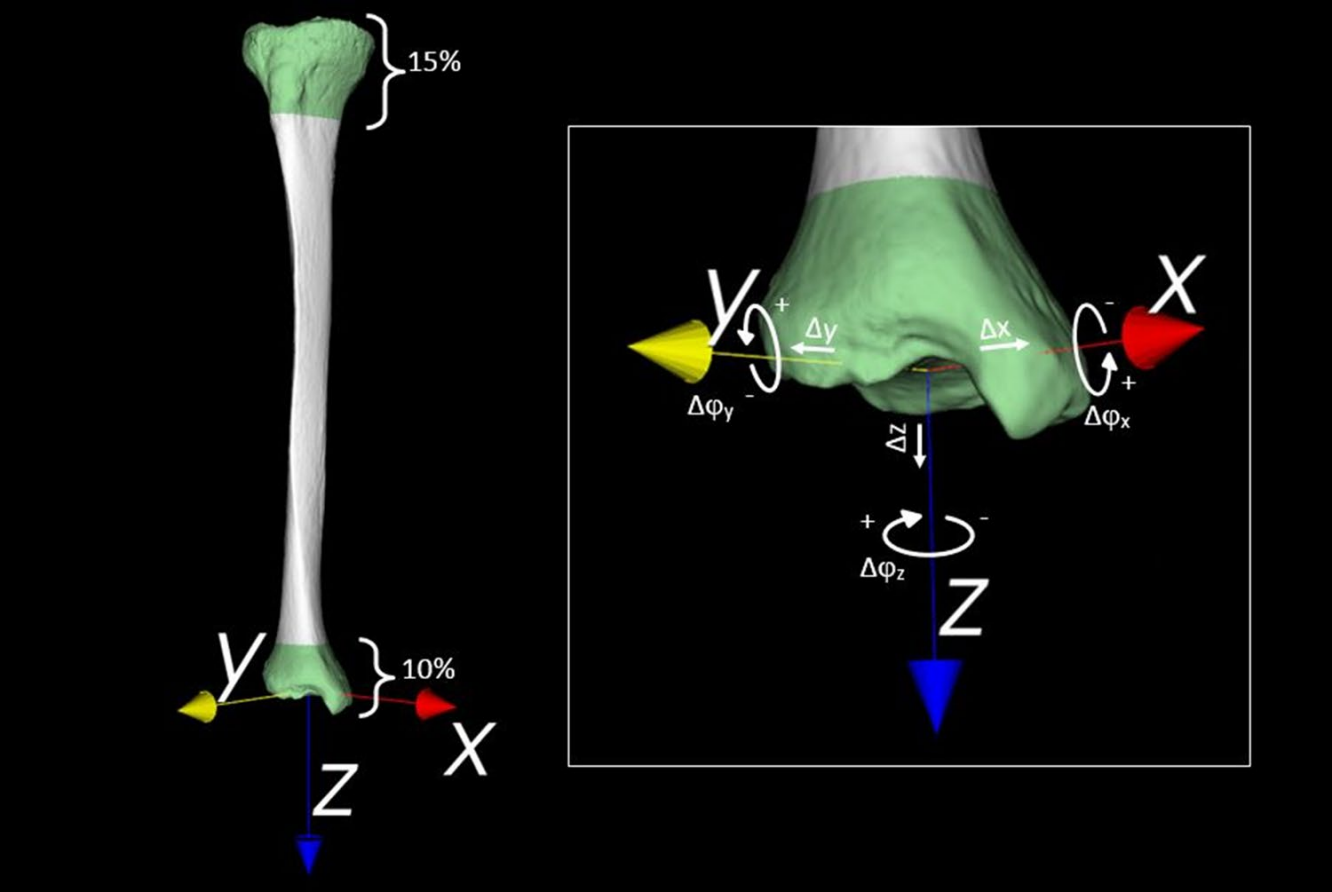
Proximal and distal segments (green) that were used to match the proximal and distal tibial segments to the contralateral side. Alignment parameters are represented by translations (Δx, Δy, Δz) along, and rotations (φ_x_, φ_y_, φ_z_) about the axes of the anatomical coordinate system, as visualized in the enlarged image. NB. The *X*-axis is in a medial–lateral direction, the *Y*-axis is in an anterior–posterior direction, the *Z*-axis is in a cranial-caudal direction (i.e., shorter/longer). φ_x_ represents a flexion/extension angulation, φ_y_ represents a varus/valgus angulation, and φ_z_ represents endotorsion/exotorsion

Alignment of the left and right tibiae was quantified by first aligning the left and right tibiae proximally, by registration. The relative position of a distal segment with respect to the proximal tibia was then used to find translational and rotational alignment parameters. This procedure effectively required registration of a proximal and distal bone segment. To this end, a proximal segment of 15% of the total tibia bone length and a distal segment of 10% of the total tibia bone length were selected from the 3D polygon. These segment lengths were pragmatically chosen and included the majority of the bone epiphysis, which helps matching polygons efficiently (Fig. 1). The distal and proximal segments were registered to the mirrored image of the other side using a point-to-image registration algorithm and provided the three translations (Δx, Δy, Δz), and three rotations (Δφ_x_, Δφ_y_, Δφ_z_) of the distal segment, which are expressed in terms of an anatomical coordinate system [4, 30].

The anatomical coordinate system was determined automatically based on the polygon mesh, as described by de Roo et al. In short: the inertia tensor is calculated based on the points of the polygon mesh of the proximally aligned right tibia [31]. Eigenvector analysis yields three eigenvectors. The vector with the smallest eigenvalue points in the direction of the bone axis and serves as *Z*-axis. The *X*-axis is perpendicular to the *Z*-axis and points to the lowest point of the polygon mesh as measured along the *Z*-axis, this is approximately in the direction of the medial malleolus. The *Y*-axis points perpendicular to the *X*- and *Z*-axes in agreement with the definition of a right-handed coordinate system.

#### Tibiofibular length ratio

The fibula has an important role specifically in the stability during mobility tasks of the ankle. Additionally, the fibula may be used as a reference standard for the initial pre-injury tibia length in case of a strong correlation between the tibia and fibula, which is expected based on previous research on the radius and ulna [20]. Therefore, the bilateral length difference was calculated, providing the tibiofibular length ratio (ΔZ_fibula_/ΔZ_tibia_).

### Statistical analysis

After checking for normality, the mean and standard deviation (SD) were calculated. To provide additional information on the symmetry parameters also, the ranges were determined. The degree of intra-individual bone symmetry was assessed using the paired Student’s *t* test comparing geometric and alignment parameters in case of a normal distribution and the Wilcoxon signed-rank test in the case of skewed data. A one-sample *t* test was used to assess whether intra-individual differences significantly differed from ‘0’. For correlated measurements (i.e., left–right legs within individuals), mixed-models analyses were used to assess differences between men and women and Pearson’s cq. Spearman’s correlation coefficients were used to assess whether there was a difference in bone geometry comparing the left and right lower leg.

A *p* value of < 0.05 was considered statistically significant. For the statistical analyses, SPSS Statistics was used (IBM Corp. Released 2016. IBM SPSS Statistics for Windows, Version 26.0. Armonk, NY: IBM Corp).

## Results

### Population demographics

Twenty healthy volunteers were included. The mean age of the individuals in this study for men was 37.7 years (± 11.1), and for women 34.0 years (± 10.3). The mean weight was 82.7 (± 5.6) kilograms (kg) for men and 71.0 kg (± 11.6) for women. The mean height was 185 cm (± 5) for men and 173 cm (± 8) for women. Sixteen volunteers reported right leg dominance and one left leg dominance, of three leg dominance was unknown.

### Geometric parameters

**Fig. 2:**
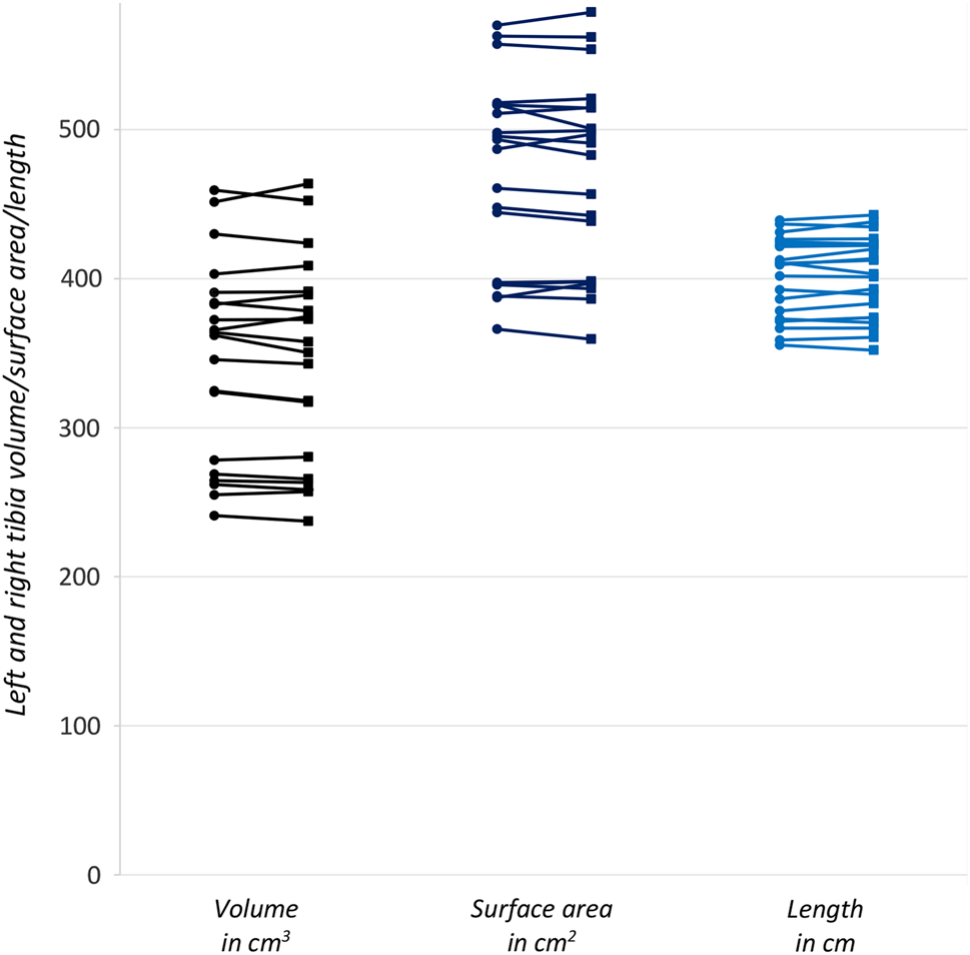
Intra-individual right-to-left difference of geometric parameters. The lines connect the left (circles) and right (squares) tibia parameter of one individual representing the intra-individual difference

Tibia volume showed largest percentage intra-individual right–left difference (1.47%), followed by bone surface (1.12%) and bone length (0.78%) subsequently (Table 1). See Fig. 2 for the intra-individual differences in geometric parameters.

**Table 1:** Tibia volume, tibia surface and tibia bone length

Tibia volume										
		Group average		Mean intra-individual difference (left leg as reference for the right leg)					Intra-individual difference (0 as reference)	
		Mean absolute tibia volume ± SD (cm^3^)	Absolute volume range (cm^3^)	Mean absolute difference ± SD (cm^3^)		Range absolute difference (cm^3^)	Relative mean difference (%)	*p* value	Mean absolute difference ± SD (cm^3^)	*p* value
Full cohort (*n* = 20)		345.8 ± 65.9	237.2–463.7	5.2 ± 3.3		0.2; 12.2	1.5	< 0.005	1.4 ± 6.1	0.326
Men vs. women	♂	391.1 ± 39.1	342.8–463.7	3.2 ± 1.3	6.8 ± 3.5	0.2; 12.2	1.9	N.s. (0.28)	1.2 ± 7.9	N.s. (0.64)
	♀	300.5 ± 55.2	237.2–408.6		3.6 ± 2.1	0.4; 6.8	1.2		1.5 ± 4.0	N.s. (0.26)

Tibia surface										
		Group average		Mean intra-individual difference (left leg as reference for the right leg)					Intra-individual difference (0 as reference)	
		Mean absolute tibia surface ± SD (cm^2^)	Absolute surface range (cm^2^)	Mean absolute difference ± SD (cm^2^)		Range absolute difference (cm^2^)	Relative mean difference (%)	*p* value	Mean absolute difference ± SD (cm^2^)	*p* value
Full cohort (*n* = 20)		468.3 ± 62.9	359.4–578.7	5.2 ± 3.3		0.6; 16.1	1.1	< 0.005	2.3 ± 6.3	N.s. (0.12)
Men vs. women	♂	516.8 ± 35.4	456.6–578.7	2.3 ± 1.8	6.3 ± 4.6	0.6; 16.1	1.2	N.s. (0.21)	2.06 ± 7.9	N.s. (0.43)
	♀	423.0 ± 47.6	359.4–514.8		4.0 ± 2.7	0.7; 9.6	1.0		2.5 ± 4.4	N.s. (0.11)

Tibia length										
		Group average		Mean intra-individual difference (left leg as reference for the right leg)					Intra-individual difference (0 as reference)	
		Mean absolute tibia length ± SD (cm)	Absolute length range (cm)	Mean absolute difference ± SD (cm)		Range absolute difference (cm)	Relative mean difference (%)	*p* value	Mean absolute difference ± SD (cm)	*P* value
Full cohort (*n* = 20)		40.2 ± 2.6	35.2–44.3	0.3 ± 0.2		0; 0.8	0.77	< 0.005	1.0 ± 3.9	N.s. (0.26)
Men vs. women	♂	41.8 ± 1.8	37.1–44.3	0.1 ± 0.1	0.4 ± 0.3	0.1; 0.8	0.8	N.s. (0.52)	1.2 ± 4.5	N.s. (0.44)
	♀	38.6 ± 2.4	35.2–43.7		0.3 ± 0.2	0.1; 0.7	0.7		0.9 ± 3.6	N.s. (0.43)

Volume, surface and length units are in cm^3^, cm^2^ and cm, respectively

*MD* mean difference, *SD* standard deviation

At group level, males (391 cm^3^ ± 39) had a significantly greater bone volume compared to women (301 cm^3^ ± 55). Gender did not influence any of the other geometric parameters.

#### Volume

The mean tibia bone volume, not taking gender or side into account, was 345.8 cm^3^ (SD ± 65.8). In seven cases, the right tibia had a greater bone volume compared to the left tibia. The absolute intra-individual volume difference ranged from 0.2 to 12.2 cm^3^ in men and 0.4–6.8 cm^3^ in women (Table 1).

#### Surface

The mean tibial surface area was 468.3 cm^2^ (SD ± 62.9). The intra-individual right–left tibia surface difference ranged from 0.6 to 16.1 cm^2^ in men and 0.7 to 9.6 cm^2^ in women (Table 1). In eight cases, the right tibial bone surface was larger compared to the left side.

#### Length

The mean tibial length was 40.2 cm (SD ± 2.7). The absolute intra-individual tibia length difference ranged from 0.1 to 0.8 cm in men and 0.1–0.7 cm in women (Table 1). In 13 cases, the right tibial bone length was larger compared to the left side.

### Alignment parameters

#### Translation

**Fig. 3:**
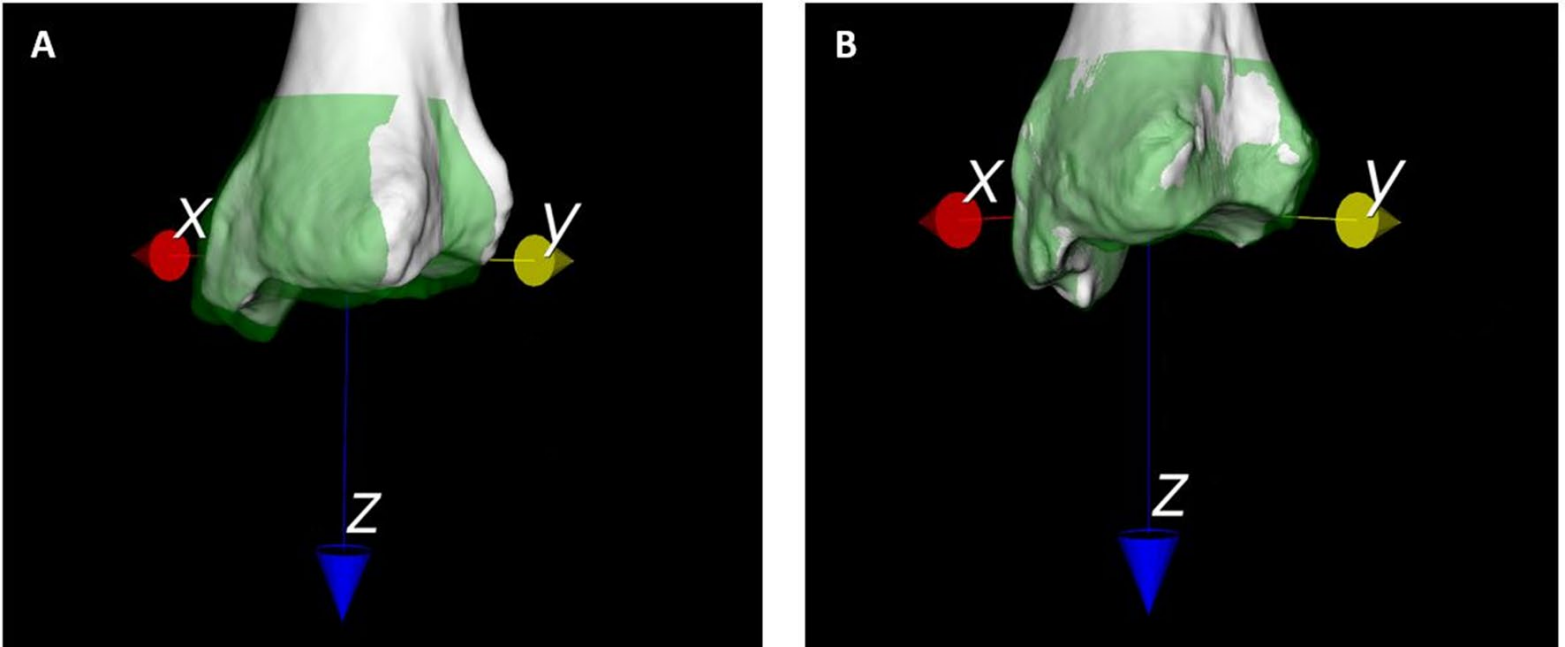
Distal matched segments of two individuals with **A** the most asymmetrical right (white) and left (green) tibiae and **B** the most symmetrical tibiae on a dorsolateral view based on intra-individual translational and rotational differences

After subsequent mirroring and matching of tibiae, intra-individual absolute translations ranged from 1.4 to 26.2 mm in men and 0.5–34.3 mm in women for the *X*-axis, 0.5–18.2 mm in men and 0.5–30.1 mm in women for the *Y*-axis, and 1.5–8.5 mm in men and 0.0–15.7 mm in women for the *Z*-axis (Table 2). Groupwise, translation expressed along the *X*-, *Y*- and *Z*-axes (Fig. 3), respectively, indicated no significant shifts along any of the axes.

**Table 2:** Mean intra-individual translation of the distal segment along the *X*-, *Y*- and *Z*-axes with respect to the proximal bone segment

Translation parameters							
			Mean intra-individual difference (left leg as reference for the right leg)			Intra-individual difference (0 as reference)	
			Mean absolute difference ± SD (mm)	Range absolute difference (mm)	Relative mean difference (%)	Mean difference ± SD (cm)	*p* value
Δx	Full cohort (*N* = 20)		9.3 ± 8.9	0.5–26.2	21.1	5.1 ± 12.1	N.s. (0.08)
	Men vs. women	♂	7.8 ± 7.1	1.4–26.2	16.6	3.9 ± 10.4	N.s. (0.27)
		♀	8.1 ± 6.8	0.5–34.3	25.6	6.3 ± 14.1	N.s. (0.19)
Δy	Full cohort		7.1 ± 7.0	0.5–30.1	20.4	1.0 ± 10.1	N.s. (0.67)
	Men vs. women	♂	5.0 ± 5.0	0.5–18.2	14.0	0.5 ± 7.4	N.s. (0.84)
		♀	9.5 ± 7.9	0.5–30.1	26.7	1.4 ± 12.7	N.s. (0.73)
Δz	Full cohort		3.9 ± 3.4	0.0–15.6	1.0	0.5 ± 5.3	N.s. (0.68)
	Men vs. women	♂	3.5 ± 2.0	1.5–8.5	0.8	0.4 ± 4.2	N.s. (0.76)
		♀	4.3 ± 4.4	0.0–15.6	1.2	1.4 ± 6.3	N.s. (0.50)

Rotation parameters						
			Mean intra-individual difference (left leg as reference for the right leg)		Intra-individual difference (0 as reference)	
			Mean absolute difference ± SD (degrees)	Range absolute difference (degrees)	Mean difference ± SD (cm)	*p* value
Δφ_x_ (flexion/extension)	Full cohort (*N* = 20)		1.3^o^ ± 1.0^o^	0.1–4.1	0.4 ± 1.7	N.s. (0.26)
	Men vs. women	♂	1.4^o^ ± 0.9^o^	0.4–3.6	0.8 ± 1.6	N.s. (0.18)
		♀	1.2^o^ ± 1.1^o^	0.1–4.1	0.1 ± 1.7	N.s. (0.84)
Δφ_y_ (varus/valgus)	Full cohort (N = 40)		1.7^o^ ± 1.4^o^	0.2–5.3	1.2 ± 1.9	0.01
	Men vs. women	♂	1.9^o^ ± 1.6^o^	0.2–3.3	1.4 ± 2.2	N.s. (0.08)
		♀	1.6^o^ ± 1.2^o^	0.4–4.2	1.1 ± 1.7	N.s. (0.08)
Δφ_z_ (endo-/exotorsion)	Full cohort (N = 40)		4.0^o^ ± 2.7^o^	0.7–10.8	2.3 ± 4.3	0.03
	Men vs. women	♂	3.9^o^ ± 3.0^o^	0.7–10.8	2.1 ± 4.7	N.s. (0.20)
		♀	4.2^o^ ± 2.4^o^	0.9–8.4	2.6 ± 4.2	N.s. (0.08)

The relative difference of the axes translation was defined based on the total axis length

*Δx* lateral translation, *Δy* anteroposterior translation, *Δz* vertical translation, *Δφ*_*x*_ rotation around the *X*-axis (flexion/extension), *Δφ*_*y*_ rotation around the *Y*-axis (varus/valgus), *Δφ*_*z*_ rotation around the *Z*-axis (endotorsion/exotorsion)

#### Rotation

The intra-individual absolute rotations expressed around the *X*- *Y*- and *Z*-axes, described by Δφ_x_/Δφ_y_/Δφ_z_ respectively, ranged from 0.4 to 3.6° in men and 0.1–4.1° in women for Δφ_x_, 0.0–5.3° in men and 0.4–4.2^°^ in women for Δφ_y_, and 0.6–12.6° in men and 0.3–8.4° in women for Δφ_z_ (Table 2). With zero as reference standard for rotational symmetry for both varus/valgus and endotorsion/exotorsion significant left-to-right differences were found.

### Tibiofibular length ratio

**Fig. 4:**
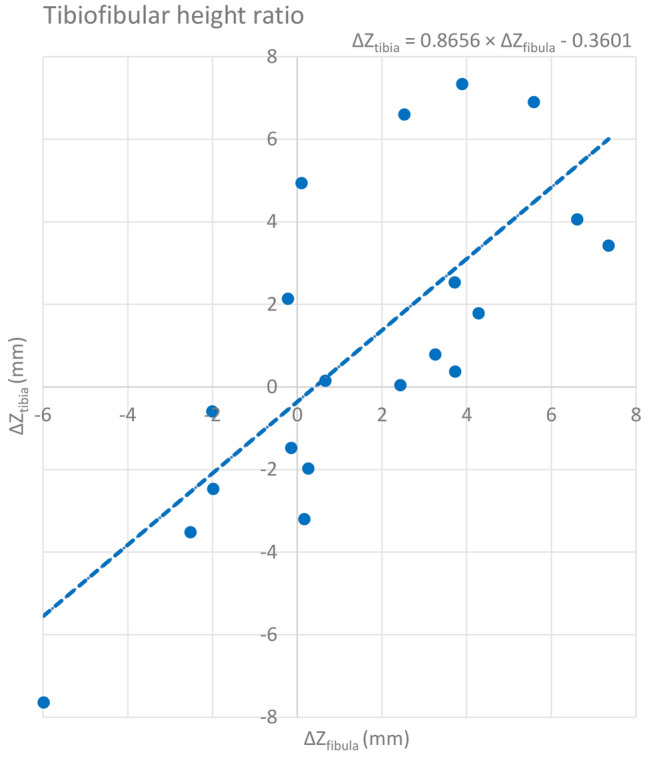
Scatterplot showing the relation between tibial length (ΔZ_tibia_) and fibular length (ΔZ_fibula_)

The mean tibial length was 40.0 cm (SD ± 2.6). The mean fibular length was 39.3 cm (SD ± 2.9). Comparing the length of the tibia in relation to the length of the fibula showed no significant differences within individuals. The Pearson’s correlation coefficient showed a strong relation between the absolute length of the tibia and the length of the fibula (*r* 0.95, *p* < 0.005) and between the mean intra-individual difference of tibia length and fibula length (ΔZ_tibia_/ΔZ_fibula_; *r* 0.75, *p* < 0.005) (Fig. 4).

### Confounders in bone symmetry

**Fig. 5:**
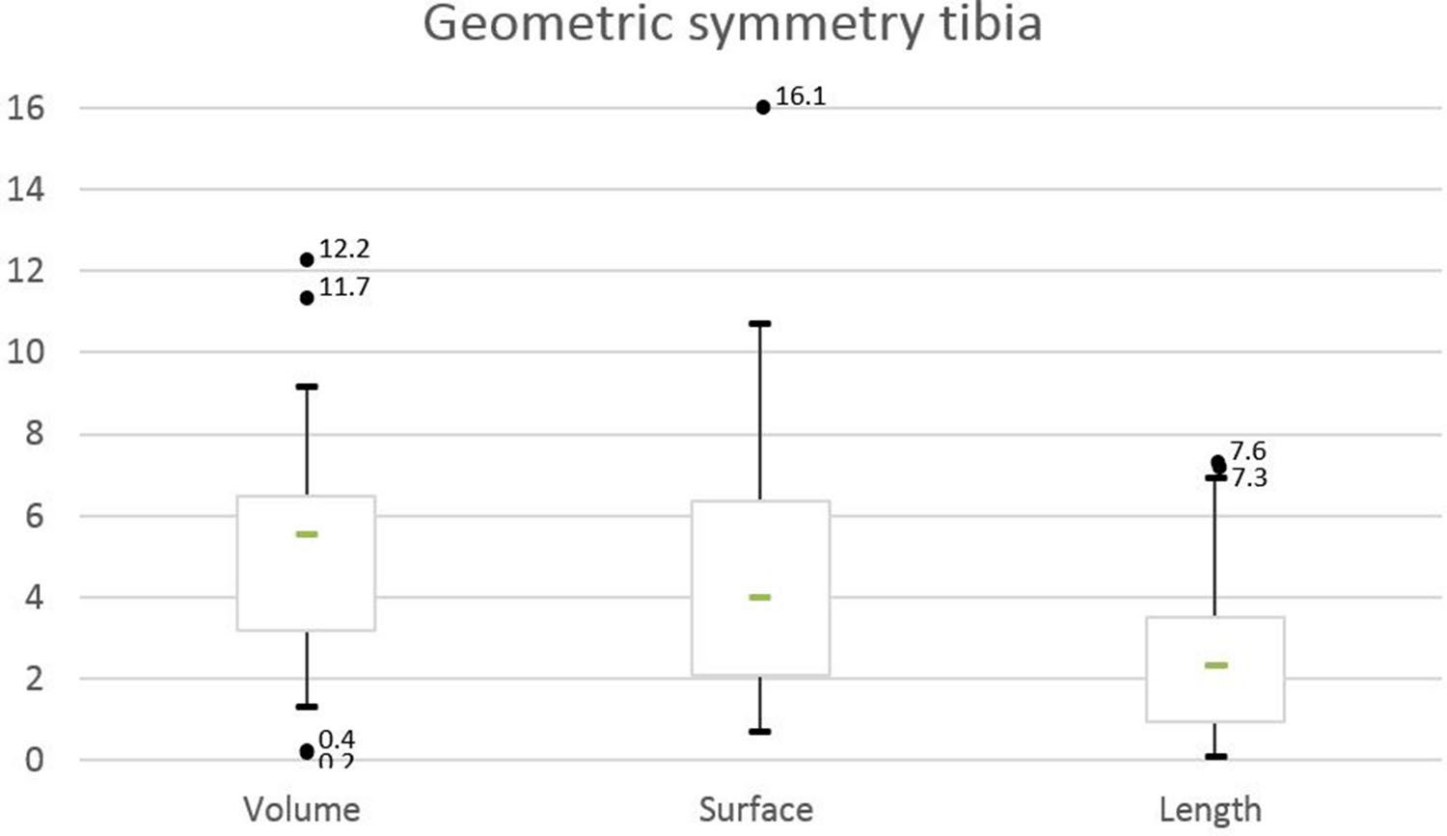
Boxplot for geometric parameters

**Fig. 6:**
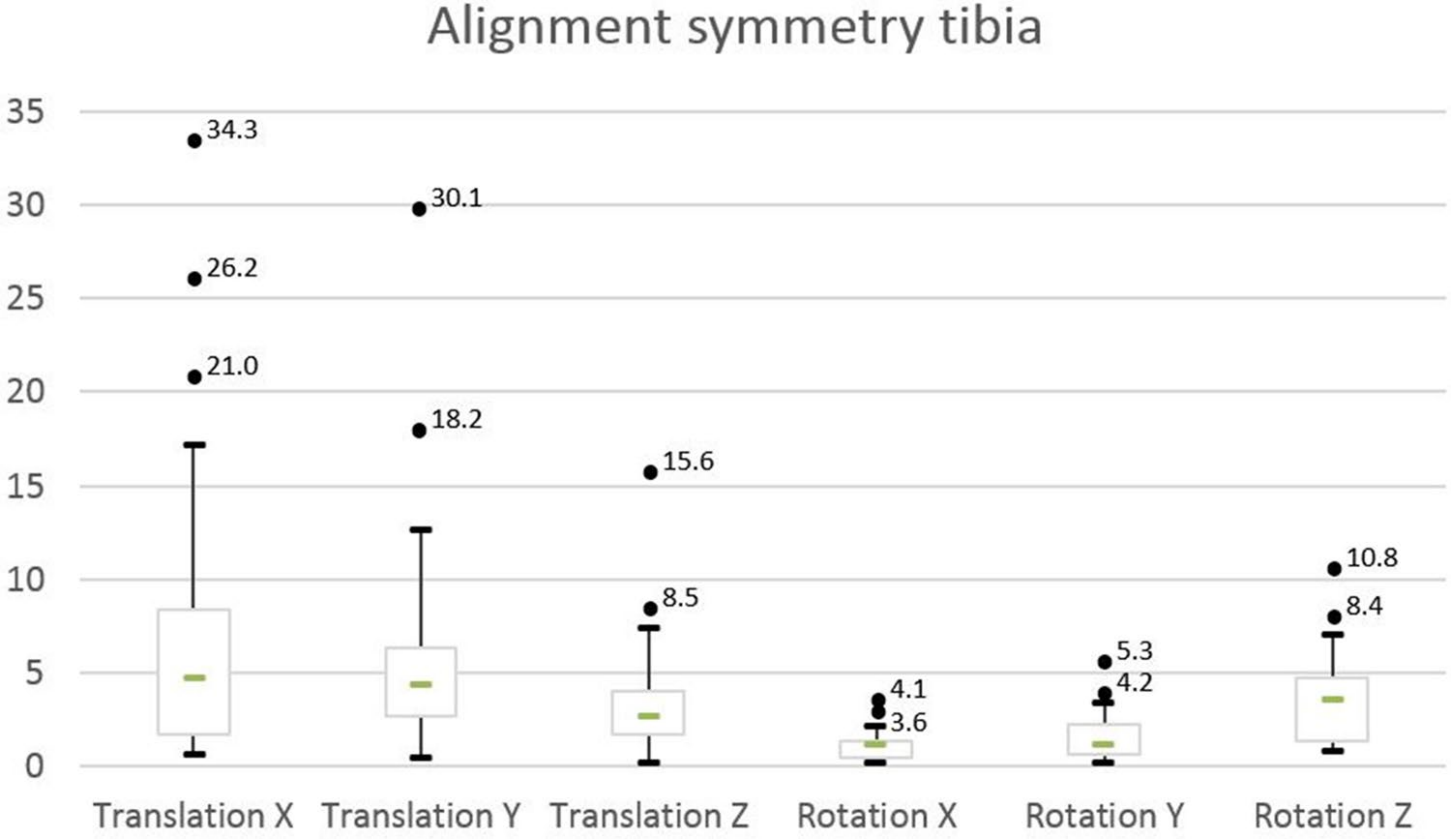
Boxplot for alignment parameters

When defining bilateral symmetry, our data indicated for both quantification of the geometric and alignment symmetry multiple outliers. For geometric bone symmetry parameters, outliers were mainly seen for bone volume (see Figs. 5, 6). There was no evident statistic relation between symmetry parameters and age, nor was either parameter more pronounced for the left or right side. For leg dominance, statistical assessment was not possible as only one volunteer reported left-leg dominance.

## Discussion

In this study, we quantified the tibial symmetry in 20 healthy individuals using geometric and alignment parameters. Groupwise, significant absolute intra-individual geometric differences were found, although these differences were smaller than two percent of the tibia volume, surface area and length. For tibia alignment, both translation and rotation differences were found. Gender did not affect the degree of symmetry. The strong correlation with fibula length may be used in the pre-operative planning to correct for tibia length differences [32]. The intra-individual differences are generally small, compared to some post-traumatic deformities. There were, however, outliers in whom the intra-individual asymmetry was greater. In these cases, the varus/valus and endotorsion/exotorsion position of the tibia was greater affecting the dynamics in the tibiotalar joint. As these patients have developed their motion pattern based on their bone geometry, post-traumatic correction may work disadvantageous as it will require patients to adapt to this correction leading to new loading patterns in the full chain (including also the knee and hip). It is yet unknown if the bilateral differences are of any clinical importance when the contralateral side is used as template for corrective surgery. Future clinical research is needed to answer this important question.

The clinical relevance of the present study is underlined by the provided novel insights into the presence or absence of bilateral symmetry in healthy volunteers. We found that there is an intra-individual tibia asymmetry in both geometric and alignment parameters. This information is important to take into account in fracture and realignment surgery as well as arthroplasty surgery. The current findings may function as a clinical and research stepping stone to identify the thresholds above which asymmetry parameters are of clinical relevance.

Previous 3D studies performed on tibial bone asymmetry by Eckhoff et al. and Schenk et al. indicated that bones did not significantly differ regarding translational parameters, but significant asymmetry existed for rotational parameters of the aforementioned bones [1, 12]. Our data, however, indicated intra-individual differences for both translation and rotation parameters. To ensure consistency and reliability of results, we assigned the coordinate system automatically instead of manually [12]. For Schenk et al., it was unclear whether the coordinate system was assigned manually or automatically [1, 27]. Additionally, we included geometric parameters to further define bone asymmetry and only included healthy volunteers, ensuring a reliable baseline in contrast to studies using cadavers with an unknown medical history [1, 7, 11, 12, 14]. Compared to Vroemen et al., who also compared asymmetry parameters in terms of translation and rotation of the distal radius and ulna, comparable right–left differences were found, although the range of left–right differences was larger, potentially caused by the larger tibia dimensions [20].

Although the findings of the aforementioned studies in addition to the present study suggest that the contralateral side may be utilized as a template concerning alignment features when performing (re) alignment surgery, there is an evident degree of asymmetry within individuals. To what degree this bilateral asymmetry may affect clinical outcome is unknown. It is necessary for future clinical studies to investigate the relation between residual malalignment and patient satisfaction in which the contralateral side is used as reference bone. In addition, as not only the tibia is paramount in leg function, also the effect of joint alignment requires more attention. Future studies will need to assess the tibiotalar and subtalar joint in light of alignment and bilateral symmetry to ensure optimal function after an ankle arthrodesis or arthroplasty.

The major strength of the current study, compared to previous research is that the current study included a full three-dimensional in vivo analysis of geometrical and alignment differences of the tibia within healthy individuals (left–right differences) instead of using a 2D projection.

Although our semi-computerized matching procedure may be the optimal procedure for assessing bone symmetry and minimized bias through manual measurements, we still had to cope with limitations. Due to the small sample size, small differences in certain bony landmarks, such as bowing of the bone leading to manual misplacement of the bone before the automatic matching procedure, may have created a variation in matching results and therefore simulated bone asymmetry. Also, our results are limited to only the tibia and fibula. The talus and calcaneus were not taken into consideration, limiting our conclusion to bone osteotomy and not being able to take lower-leg function and joint alignment into consideration.

In conclusion, this study quantifies small but significant intra-individual tibia asymmetry in both geometric and alignment parameters. The surgeon needs to be aware of these differences, especially the existence of outliers, when using the contralateral side as reference for realignment planning. However, the ranges in intra-individual asymmetry are on average below two percent and may therefore not be clinically relevant. Intra-individual symmetry was greatest for tibia surface and length, and in the sagittal plane (flexion/extension). The high correlation between tibia and fibula length allows the ipsilateral fibula to aid in estimating the true tibia length in pre-operative planning. The greatest asymmetry was seen for translation, and rotation in the coronal (varus/valgus) and axial (endotorsion/exotorsion) plane.
